# A comparison of the chest radiographic and computed tomographic features of subclinical pulmonary tuberculosis

**DOI:** 10.1038/s41598-022-21016-7

**Published:** 2022-10-04

**Authors:** Angela Lau, Christopher Lin, James Barrie, Christopher Winter, Gavin Armstrong, Mary Lou Egedahl, Alexander Doroshenko, Courtney Heffernan, Leyla Asadi, Dina Fisher, Catherine Paulsen, Jalal Moolji, Yiming Huang, Richard Long

**Affiliations:** 1 The Department of Medicine, Faculty of Medicine and Dentistry, University of Alberta, Room 8325, Aberhart Centre, 11402 University Avenue, Edmonton, AB T6G 2J3 Canada; 2 The Department of Family Medicine, Faculty of Medicine and Dentistry, University of Alberta, Edmonton, AB Canada; 3 The Department of Radiology and Diagnostic Imaging, Faculty of Medicine and Dentistry, University of Alberta, Edmonton, AB Canada; 4 Cumming School of Medicine, University of Calgary, Calgary, AB Canada

**Keywords:** Respiratory tract diseases, Infectious diseases, Tuberculosis, Medical imaging

## Abstract

Subclinical pulmonary tuberculosis (PTB) is a recently described intermediate state of great interest, but about which little is known. This study sought to describe and compare the frequency of key radiologic features of subclinical PTB on chest radiograph (CXR) versus computed tomographic scan (CT), and to interpret the clinical and public health relevance of the differences. Diagnostic CXRs and CT scans of the thorax and neck in a 16-year cohort of subclinical PTB patients in Canada were re-acquired and read by two independent readers and arbitrated by a third reader. Logistic regression models were fit to determine how likely CXR features can be detected by CT scan versus CXR after adjustment for age and sex. Among 296 subclinical patients, CXRs were available in 286 (96.6%) and CT scans in 94 (32.9%). CXR features in patients with and without CT scans were comparable. Lung cavitation was 4.77 times (95% CI 1.95–11.66), endobronchial spread 19.36 times (95% CI 8.05–46.52), and moderate/far-advanced parenchymal disease 3.23 times (95% CI 1.66–6.30), more common on CT scan than CXR. We conclude that the extent to which CXRs under-detect key radiologic features in subclinical PTB is substantial. This may have public health and treatment implications.

## Introduction

Subclinical tuberculosis (TB) is one of two recently described intermediate states, the other being incipient TB, that occur between TB infection and symptomatic pulmonary TB (PTB)^1, 2^. It has been defined as “a state of disease due to viable *Mycobacterium tuberculosis* that does not cause TB-related symptoms but does cause other abnormalities that can be detected using existing radiologic or microbiologic assays”^1, 2^. As the earliest detectable disease state, subclinical PTB has generated great interest. Its importance to public health is potentially substantial given that such patients may continue to interact with others as normal for extended periods of time before being diagnosed and treated, or spontaneously reverting to an inactive state^1–5^.

In a large representative sample of adult culture-positive PTB patients in Canada, and using a strict case definition, we found that the proportion of patients who were determined to be subclinical, i.e. literally asymptomatic, was 21.0%^6^. Most subclinical PTB patients were discovered during one or other active case-finding exercise, such as the screening of immigration referrals, the screening of close contacts of sputum smear-positive clinical patients, or the screening of extrapulmonary TB patients for evidence of concomitant PTB^7–10^. Only 11.2% were found to be smear-positive and the vast majority (86.4%) of those who were HIV-seronegative and did not have relapse/retreatment disease (i.e. they had new-active disease) had either no or minimal lung parenchymal disease on chest radiograph^6^. The present study is an extension of this earlier study. Herein we seek to measure the extent to which the chest radiograph under-detects key radiologic features, in particular cavitation and extent of parenchymal disease in subclinical PTB. To do so we determined how likely a range of radiographic features can be detected using computed tomography versus the chest radiograph.

## Methods

Institutional approval was obtained from, and informed consent was waived by, the Health Research Ethics Board of the University of Alberta, proposal ID: PRO00108560. We performed a retrospective cohort study wherein we identified all adults (age > 14 years) who were diagnosed with culture-positive PTB in the Province of Alberta, Canada (population 4,464,170 in 2021 [Statistics Canada]), between January 1, 2005 and December 31, 2020^6^. Alberta Precision Laboratories, where all mycobacteriology in the province is performed, confirmed the culture status of each patient. Patients’ symptom status, i.e. whether they had one or more TB-compatible respiratory (cough [dry or productive], hemoptysis, chest pain or dyspnea), or constitutional/systemic (weight loss, fever, night sweats, fatigue) symptom had been recorded by the public health nurse and/or the attending physician, at the time of diagnosis. Final determination of symptomatic versus asymptomatic was achieved through independent review of public health and hospital records by two members of the study team. Discrepancies were resolved by consensus. Patients with extrapulmonary TB who had no respiratory symptoms were considered asymptomatic for PTB, though they may have had symptoms localized to their other site(s) of disease.

Symptomatic (hereafter ‘clinical’) and asymptomatic (hereafter ‘subclinical’) PTB patients were described according to age, sex, country of birth (Canadian-born vs foreign-born), and mycobacteriologic features. The latter included smear status, average time-to-liquid-culture positivity of pre-treatment sputum or other airway secretion specimens; i.e. those specimens collected on or up to 8 weeks before the start date of treatment, and first-line anti-TB drug resistance. Laboratory cross-contamination was systematically excluded in all patients with a single smear-negative culture-positive specimen^11^. Patients who were HIV-seropositive or HIV-unknown and patients with relapse/retreatment disease or whose disease type was unknown, were excluded due to the potential impact HIV coinfection and/or past TB may have on the chest radiograph. Finally, subclinical patients were further described according to their reason-for-assessment: (1) immigration referral if foreign-born including immigrants, refugees, and some temporary residents whose admission to Canada is conditional on compliance with TB surveillance; (2) a concomitant diagnosis of extrapulmonary TB; (3) positive tuberculin skin test (TST) or interferon-gamma release assay (IGRA) identified during contact tracing, employment screening, or screening of patients at increased risk of developing reactivation TB if infected; or, (4) miscellaneous other.

The name, date of birth and personal health care number of subclinical PTB patients was used to identify records in the provincial picture archiving and communication system (PACs). The date of the posterior-anterior (PA) and lateral (LAT) chest images closest to the start date of treatment were recorded and images acquired. The dates of all computed tomographic (CT) scans of the thorax, or neck in those with cervical lymph node TB, performed in close proximity to the diagnostic chest radiograph were recorded and images acquired. Data abstraction forms were used to report and categorize the chest radiograph and CT scan features of PTB (see Supplement 1 and 2)^12^. Images were independently read by two chest radiologists (with 25 and 15 years of experience, respectively); radiographic interpretations were blind to CT interpretations. Discrepant interpretations were resolved by a third chest radiologist (with 15 years of experience).

### Chest radiographic features

i) Category: radiographic findings were categorized as ‘typical’ for adult-type PTB (upper lung zone predominant disease, with or without cavitation, but no discernable adenopathy), ‘atypical’ for adult-type PTB (abnormalities inconsistent with the definition of typical), or normal. Lung zones were determined by visualizing a perpendicular line from the apex of the lung to the hemidiaphragm and dividing the lung in half; the superior segment of the lower lobe was considered part of the upper lung zone.
ii) Laterality: bilateral, unilateral, or normal.
iii) Cavitation: cavitary or non-cavitary disease (a cavity defined as a gas filled space within pulmonary consolidation, a mass or a nodule); whether cavities were single or multiple and the internal diameter of the largest cavity.
iv) Endobronchial spread: acinar shadows (multiple ill-defined nodules 4–8 mm in diameter).
v) Adenopathy: enlarged hilar and/or mediastinal lymph nodes.
vi) Extent of parenchymal disease: normal, minimal, moderately-advanced, far-advanced, or miliary according to criteria established by the US National Tuberculosis and Respiratory Disease Association (see supplement 3)^13^. In this definition, the presence of cavitation automatically infers either moderately- or far-advanced disease.

Incidental note was also made of the presence of volume loss, pleural thickening/retraction or effusion. If adenopathy alone was identified, i.e. the lung parenchyma appeared normal, the category was reported as atypical and the laterality and extent of disease were reported as normal. When the only abnormality was a single small (< 1 cm) calcified nodule (granuloma), the radiograph was reported as normal. Normal radiographs included both PA and LAT views.

### Computed tomographic features

i) Category: typical, atypical, or normal as per the chest radiograph definition with the exception that when the only abnormality was enlarged lymph nodes and enlarged lymph nodes had not been reported on the corresponding chest radiograph the scan was reported as typical.
ii) Laterality: unilateral or bilateral or normal.
iii) Endobronchial spread: either centrilobular nodules or opacities (well defined lesions 2 to 4 mm separated by > 2 mm from the pleural surface or interlobular septa) with or without “tree-in-bud” (a branching linear structure with more than one contiguous branching site) or poorly defined nodules 5–8 mm in diameter^14^, or both.
iv) Cavitation: as per the chest radiograph, whether single or multiple, the internal diameter of the largest cavity.
v) Adenopathy: hilar or mediastinal lymph node enlargement defined as a node > 10 mm short axis diameter.
vi) Extent of parenchymal disease: normal, minimal, moderately-advanced, far-advanced as per the chest radiograph definition; determination to include review of coronal sections.

The scan type (thorax or neck), date of performance, and parameters (i.e. enhanced or non-enhanced; collimation) were recorded. A CT of the neck was accepted in cervical lymph node TB patients if, (i) it extended to the main carina, and (ii) the only abnormality, if any, on the corresponding chest radiograph was above the level of the main carina. As per the chest radiograph, note was also made of the presence or absence of volume loss, pleural thickening/retraction or effusion.

### Statistical analysis

To assess differences between the demographic and mycobacteriologic features in subclinical and clinical patients, we used chi square or Fisher’s exact test for categorical variables and t-test for continuous variables. To quantify the level of agreement between readers for radiographs and CT scans, we report generalized kappa statistics, and for the purpose of results, we report the arbitrated values. The difference in proportions of radiographic features present in patients who underwent a chest radiograph alone versus those who had both chest radiograph and CT scan were determined using chi square or Fisher’s exact test. We fit logistic regression models to determine how likely radiographic features can be detected by the CT scan versus the chest radiograph, adjusted for universal confounders, age and sex. We use the conventional *p* value < 0.05 for statistical significance. Methods were performed in accordance with the relevant guidelines and regulations. All analyses were performed in STATA, version 14.0.

## Results

Over the 16 years 2005–2020, 1,656 adults were diagnosed with culture-positive PTB in Alberta; 347 (21.0%) subclinical, 1309 (79.0%) clinical. Of the 347 subclinical patients, 296 (85.3%) were HIV-seronegative and new-active; of the 1309 clinical patients, 1095 (83.7%) were HIV-seronegative and new-active. Compared to HIV-seronegative new-active clinical PTB patients, HIV-seronegative new-active subclinical PTB patients were more likely to be foreign-born (90.5% vs 80.6%), smear-negative (90.5% vs 43.4%), and have a longer time-to-culture positivity (median [IQR] 18.0 [14.5–25.0] vs 11.9 [7.0–17.0] days), see Table 1. Almost half of the subclinical patients (144 or 48.6%) had been assessed as immigration referrals. Of those who were assessed because of concomitant extrapulmonary TB (n = 44), 35 (79.5%) had cervical lymph node TB. Of those who were assessed because of a positive TST/IGRA (n = 35), 21 (60.0%) were contacts of smear-positive clinical PTB. The remaining subclinical patients (n = 73 or 24.7%) had their PTB identified incidentally during work-up of other conditions such as cancer or trauma.

**Table 1:** Demographic and mycobacteriologic features in HIV-seronegative, new-active pulmonary tuberculosis patients by symptom status; reason for assessment of subclinical patients

Features	Total	PTB patients		*p* value
		Subclinical No (%)	Clinical No (%)	
**No. assessed**	1391	296	1095	
**Age (years)**				0.098
15–64	1009 (72.5)	226 (76.4)	783 (71.5)	
> 64	382 (27.5)	70 (23.6)	312 (28.5)	
**Sex**				0.066
Male	789 (56.7)	154 (52.0)	635 (58.0)	
Female	602 (43.3)	142 (48.0)	460 (42.0)	
**Population group**				0.00006
Canadian-born	240 (17.3)	28 (9.5)	212 (19.4)	
Foreign-born	1151 (82.7)	268 (90.5)	883 (80.6)	
**Smear status**				< 0.00001
Positive	648 (46.6)	28 (9.5)	620 (56.6)	
Negative	743 (53.4)	268 (90.5)	475 (43.4)	
**Time to culture-positivity (days)** ^†^				0.0001
Mean ± SD	14.9 ± 9.0	20.9 ± 9.1	13.3 ± 8.3	
Median (IQR)	13.3 (8.5–18.6)	18.0 (14.5–25.0)	11.9 (7.0–17.0)	
**Drug-resistance**^‡^				0.890
Yes	144 (10.4)	30 (10.1)	114 (10.4)	
No	1247 (89.6)	266 (89.9)	981 (89.6)	
**Reason for assessment**^§^				
Immigration referral		144 (48.6)		
EPTB		44 (14.9)	N/A	
Positive TST/IGRA		35 (11.8)		
Miscellaneous		73 (24.7)		

Abbreviations: HIV human immunodeficiency virus; PTB pulmonary tuberculosis; SD standard deviation; IQR interquartile range; EPTB extrapulmonary tuberculosis; TST tuberculin skin test; IGRA interferon gamma release assay; NA not applicable.

The patients reported in Table 1 are a subset of patients previously reported (see reference #6).

^†^If multiple pre-treatment specimens were culture-positive, the average time to culture-positivity was used.

^‡^Resistance to one or more first-line anti-tuberculosis drugs.

^§^See text for a full description of the reason for assessment.

Chest radiographs were available in 327 of the 337 HIV-seronegative subclinical patients (97.0%), 286 new-active and 41 relapse/retreatment. CT scans were available in 98 of the 337 HIV-seronegative subclinical patients (29.1%), 94 new-active and 4 relapse/retreatment. Inter-reader variability was assessed on all images, see Table 2. Kappa statistics for the chest radiograph showed substantial agreement (0.61–0.80) for category, laterality, lymph node enlargement, and extent of parenchymal disease, and moderate agreement (0.41–0.60) for cavitation and endobronchial spread. Kappa statistics for the CT scan showed almost perfect agreement (0.81–0.99) for category, laterality, cavitation, and extent of disease, substantial agreement for lymph node enlargement, and moderate agreement for endobronchial spread. As previously reported (6), subclinical patients with relapse/retreatment PTB were significantly more likely to be smear-positive and have more extensive disease on chest radiograph; hence their chest images were excluded from further analysis.

**Table 2:** Expert inter-reader variability of subclinical PTB chest radiograph and computed tomogram interpretation

Imaging modality	Feature^†^	Agreement	Kappa Statistic	95% CI
Chest radiograph	Category Laterality Cavitation Endobronchial Spread Lymph Node Enlargement Extent of Disease	Substantial Substantial Moderate Moderate Substantial Substantial	0.745 0.723 0.483 0.441 0.657 0.681	0.672–0.819 0.655–0.791 0.317–0.649 0.257–0.645 0.401–0.914 0.604–0.758
Computed tomogram	Category Laterality Cavitation Endobronchial Spread Lymph Node Enlargement Extent of Disease	Almost perfect Almost perfect Almost perfect Moderate Substantial Almost perfect	0.829 0.882 0.975 0.504 0.789 0.800	0.713–0.944 0.799–0.966 0.928–1.000 0.340–0.667 0.640–0.938 0.700–0.899

The kappa statistic for the chest radiograph features have been previously reported (see reference #6).

^†^ See text for definition of chest radiographic and computed tomographic features; see reference #13 and the online Supplement 3 for definition of extent of disease.

Typical adult-type PTB was present on chest radiograph in 68.2% of HIV-seronegative new- active subclinical PTB patients (82.2% of those whose radiograph was abnormal), see Table 3. Normal chest radiographs were not uncommon (17.5%); 15 or 30% of patients with a normal chest radiograph were TB contacts with a positive TST or IGRA. Bilateral disease was present in 24.5%. Cavitation and evidence of endobronchial spread (acinar shadows) were uncommon, 6.6% and 7.1%, respectively. Cavities, when present, were usually small (median internal diameter 13.0 mm; IQR 9.5–16.0 mm) and singular (73.7%). Most patients with cavitation on chest radiograph had a typical adult-type PTB pattern of disease (17/19 or 89.5%). Intrathoracic lymph node enlargement was rare (3.1%); in 2 (22.2%) patients it was associated with cervical lymph node TB. The “extent of parenchymal disease” was normal in 18.9% and minimally abnormal in 67.5%. Volume loss and pleural thickening/retraction were uncommon (15.9% and 10.6%, respectively), while pleural effusion was rare (2.8%), data not shown. Radiographic features in subclinical patients who had or had not undergone a CT scan were similar, with the exception of radiograph category which was more likely to be atypical in those who underwent a CT scan versus those who did not.

**Table 3:** Chest radiographic features of subclinical pulmonary tuberculosis in patients with and without a computed tomographic scan

Feature	Total No. (%)	Subclinical PTB Cases		*p* value
		CXR Alone No (%)	CXR and CT No (%)	
**No. assessed**	286	192	94	
**Category**				
Typical	195 (68.2)	133 (69.3)	62 (66.0)	0.037
Atypical	41 (14.3)	21 (10.9)	20 (21.3)	
Normal	50 (17.5)	38 (19.8)	12 (12.8)	
**Laterality**				
Unilateral disease	162 (56.6)	106 (55.2)	58 (61.7)	0.438
Bilateral disease	70 (24.5)	46 (24.0)	22 (23.4)	
Normal	54 (18.9)	40 (20.8)	14 (18.9)	
**Cavitation**				
Yes	19 (6.6)	12 (6.3)	7 (7.4)	0.703
No	267 (93.3)	180 (93.8)	87 (92.6)	
**Acinar shadows**^†^				
Yes	19 (6.7)	12 (6.9)	7 (7.4)	0.728
No	264 (93.3)	177 (93.7)	87 (92.6)	
**Lymph node enlargement**				
Yes	9 (3.1)	4 (2.1)	5 (5.3)	0.16
No	277 (96.9)	188 (97.9)	89 (94.7)	
**Extent of parenchymal disease**				
Normal	54 (18.9)	40 (20.8)	14 (14.9)	0.179
Minimal	193 (67.5)	131 (68.2)	62 (66.0)	
Moderately advanced	37 (12.9)	20 (10.4)	17 (18.1)	
Far advanced	0 (0.0)	0 (0.0)	0 (0.0)	
Miliary	2 (0.7)	1 (0.5)	1 (1.1)	

Abbreviations: PTB pulmonary tuberculosis; CXR chest x-ray; CT computed tomography.

The proportions of all patients (“total” column) with the features have been previously reported (see reference #6).

^†^ Information on acinar shadows on the chest radiograph was unavailable in three patients.

**Figure 1:**
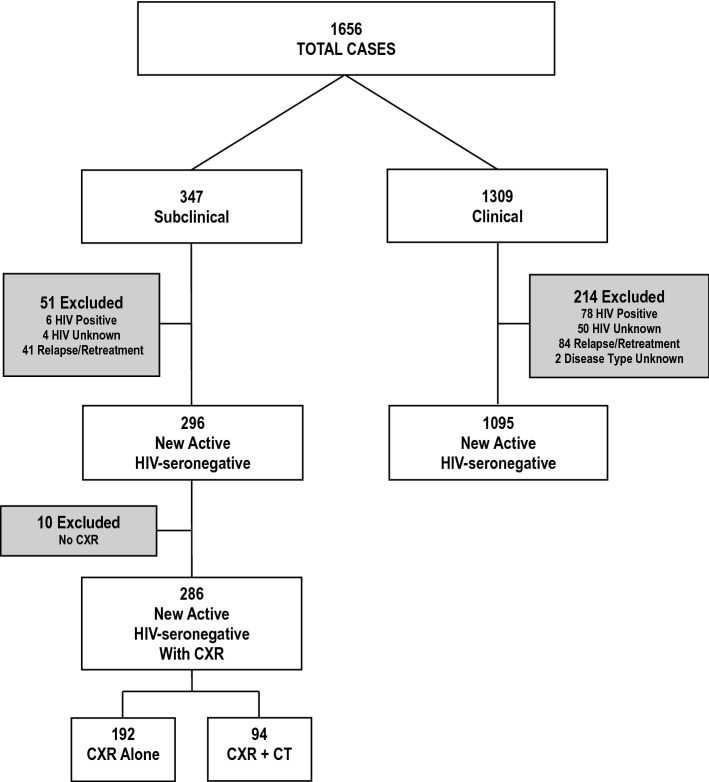
Adult culture-positive pulmonary tuberculosis patients in the Province of Alberta, Canada, 2005–2020. Chest radiographs were performed in all but 10 of the HIV-seronegative, new active subclinical patients and all 41 of the HIV-seronegative relapse/retreatment subclinical PTB patients. CT scans were performed in 94 of the 286 HIV-seronegative new active subclinical PTB patients with chest radiographs and 4 of the 41 HIV-seronegative relapse/retreatment subclinical PTB patients with chest radiographs. This figure is an adaptation and extension of a previously reported figure. (see reference #6).

**Figure 2:**
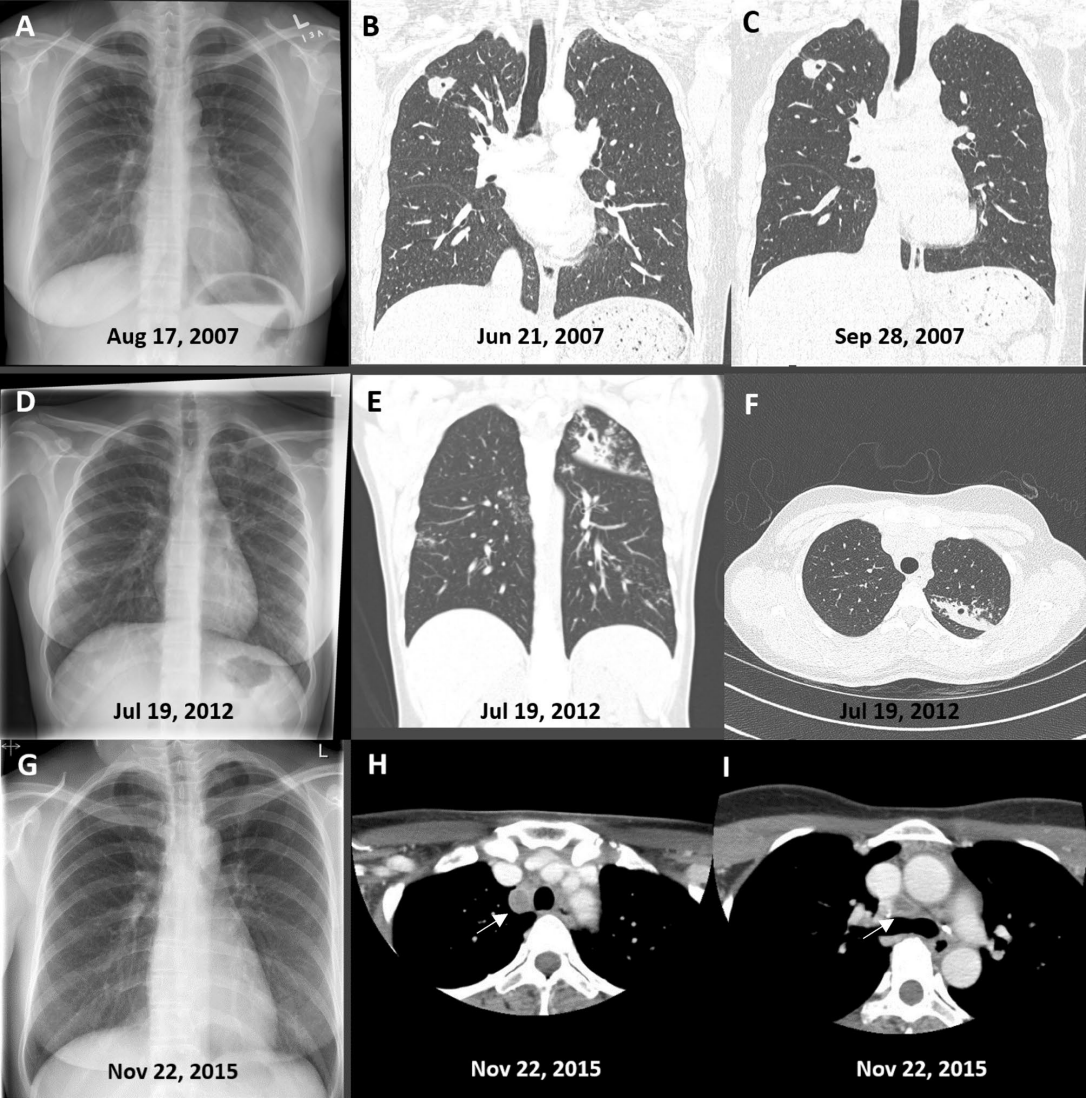
(**A,B,C**) Frontal chest radiograph (**A**) and thin Sect. (1.0 mm) coronal reformat computed tomographic (CT) images (**B** and **C**) in a patient diagnosed with smear-negative, culture-positive subclinical pulmonary tuberculosis on August 28, 2007. A small cavity is visible on CT scan but not on chest radiograph; the abnormality in the right upper lobe is stable over several months. (**D,E,F**) Frontal chest radiograph (**D**), a multiplanar reformation (MPR) coronal CT image (3 mm) (**E**) and a 1 mm transverse CT image through the upper thorax (**F**) in a patient diagnosed with smear-negative, culture-positive subclinical pulmonary tuberculosis on July 26, 2012. Cavitation is visible on CT scan but not on chest radiograph; endobronchial spread is more visible on CT scan than on chest radiograph. (**G,H,I**) Frontal chest radiograph (**G**) and 3 mm transverse CT images at the level of the aortic arch (**H**) and main carina (**I**) in a patient diagnosed with smear-positive, culture-positive subclinical pulmonary tuberculosis on November 24, 2015. An enlarged, centrally necrotic, lymph node is visible in the right paratracheal area (**H**) and a borderline enlarged centrally necrotic lymph node is visible just anterior to the main carina (**I**) on CT scan; neither was clearly visible or reported on chest radiograph.

Of the 286 HIV-seronegative, new-active subclinical patients with chest imaging, 94 (32.1%) had undergone both a chest radiograph and a CT scan (Fig. 1). The median (IQR) time between the two studies was 10 (2 to 27) days. There were 86 scans of the thorax and 8 scans of the neck. The median (IQR) collimation was 1.0 (1.0 to 2.5); 68 scans (72.3%) were thin section (≤ 2 mm); 62 (66.0%) scans were enhanced. Going from one diagnostic imaging modality to another (chest radiograph to CT scan), the odds of having bilateral disease were 1.95 times (confidence interval [CI] 1.01–3.74), cavitation 4.77 times (CI 1.95–11.66), endobronchial spread 19.36 times (CI 8.05–46.52), intrathoracic lymph node enlargement 6.56 times (CI 2.15–20.02), and extensive parenchymal disease 3.23 times (CI 1.66–6.30), higher, see Table 4 and Fig. 2. Cavities were usually small, median (IQR) internal diameter 8.5 mm (5.5–11.5 mm) and singular (65.4%). Most patients with cavitation on CT scan had “typical” adult-type disease (23/26 or 88.5%). Volume loss was present in 26.6%; pleural thickening/retraction and pleural effusion in 8.5% and 8.5%, respectively, data not shown.

**Table 4.** Detection of radiologic features among subclinical PTB patients by diagnostic imaging modality

Feature	Radiograph No (%)	CT Scan No (%)	OR (95% CI)	*p* value
**No. assessed**	94	94		
**Category**				
Typical	62 (66.0)	69 (73.4)		
Atypical	20 (21.3)	20 (21.3)	1.36 (0.72–2.55)	0.339
Normal	12 (12.8)	5 (5.3)		
**Laterality**				
Unilateral	58 (61.7)	52 (55.3)		
Bilateral	22 (23.4)	36 (38.3)	1.95 (1.01–3.74)	0.046
Normal	14 (18.9)	6 (6.4)		
**Cavitation**				
Yes	7 (7.4)	26 (27.7)	4.77 (1.95–11.66)	0.001
No	87 (92.6)	68 (72.3)		
**Endobronchial spread**				
Yes	7 (7.4)	56 (59.6)	19.36 (8.05–46.52)	< 0.001
No	87 (92.6)	38 (40.4)		
**Lymph node enlargement**				
Yes	5 (5.3)	21 (22.3)	6.56 (2.15–20.02)	0.001
No	89 (94.7)	73 (77.7)		
**Extent of parenchymal disease**				
Normal	14 (14.9)	7 (7.4)		
Minimal	62 (66.0)	47 (50.0)		
Moderately ADVANCED	17 (18.1)	35 (37.2)	3.23 (1.66–6.30)	0.001
Far advanced		1 (1.1)		
Miliary	1 (1.1)	4 (4.3)		

Abbreviations: PTB pulmonary tuberculosis; CT computed tomography; OR odds ratio; CI confidence interval.

Adjusted for age and sex.

**Figure 3:**
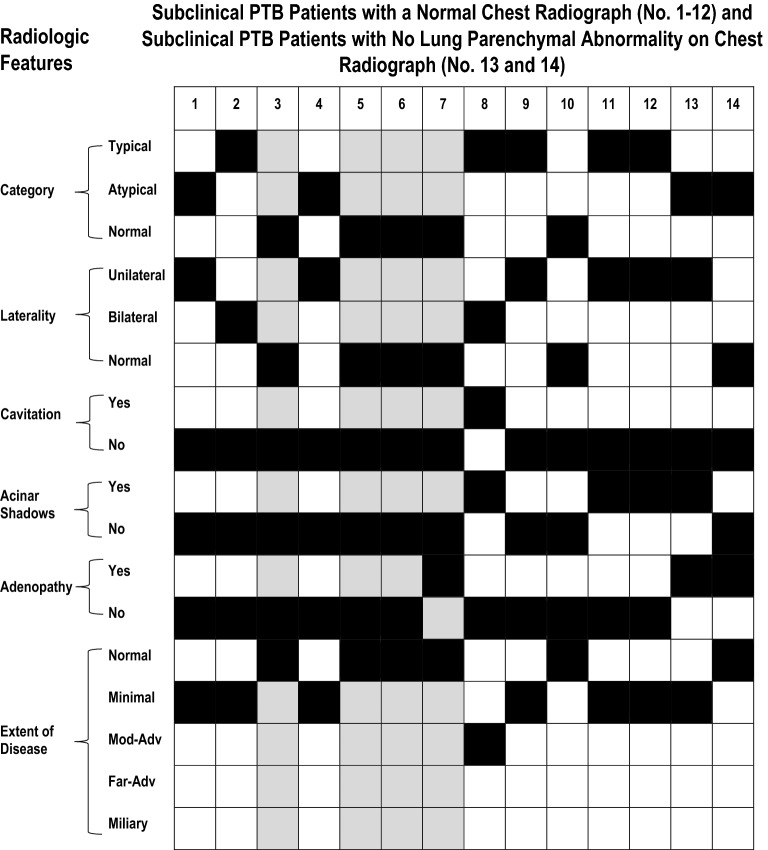
Computed tomographic (CT) scan results in patients who had normal chest radiographs (patients #1–12) and in patients whose chest radiographs were abnormal but showed no lung parenchymal abnormality (patients #13 and 14). Positive findings are indicated by a black box . Back-shaded columns (3, 5, 6, 7) refer to patients who had a CT scan of the neck. Patients #3, 5, 6, 7, and 10 had concomitant cervical lymph node TB. Patients #9 and #12 were close contacts of smear-positive clinical patients. In Patient #8 (i.e. the patient whose chest radiograph was reported as normal and whose CT was reported as showing moderately advanced cavitary disease) the chest radiograph and CT scan were performed on the same day. Abbreviations: CXR chest X-ray; MOD moderately; ADV advanced.

Of the 14 patients who had no lung parenchymal disease on chest radiograph (12 with completely normal chest radiographs, 2 with intrathoracic lymph node enlargement only), 7 had minimal and 1 had moderately-advanced disease (with cavitation) on CT scan (see Fig. 3). Endobronchial spread on CT scan was more likely in those with than those without lung parenchymal disease on chest radiograph (52/80 [65.0%] vs. 4/14 [28.6%], *p* < 0.02).

## Discussion

After careful review of clinical and public health records by two independent reviewers and exclusion of laboratory case contamination, we found, as previously reported^6^, that 21.4% of incident HIV-seronegative, new-active culture-positive PTB patients in this high-income, low incidence setting were subclinical. Most HIV-seronegative, new-active subclinical patients were foreign-born persons whose disease was discovered during one or other active case-finding exercise. They were usually AFB smear-negative (90.5%) and had, on average, long times-to-liquid-culture positivity (median [IQR] 18.0 [14.5–25.0] days). Most (86.4%) had minimal or no lung parenchymal abnormality on chest radiograph. But, importantly, as reported herein, from a public health and possibly a treatment perspective, the chest radiograph significantly under-detected key features of disease; cavitation was 4.77 times, endobronchial spread 19.36 times, and extensive lung parenchymal disease 3.23 times more likely to be present on CT scan than chest radiograph.

While many of the classical features of adult-type PTB were present on plain chest radiograph in this cohort of HIV-seronegative, new-active subclinical PTB patients, others were notably absent. Most patients whose chest radiograph was abnormal had a typical pattern of adult-type PTB – upper lung zone predominant disease, with or without cavitation, but no discernable adenopathy^15^. Cavitation on the other hand, while previously reported to be present on chest radiograph in 30–50% of adult-type PTB patients^16–18^, was present in only 6.6% of subclinical PTB patients. Likewise, endobronchial spread (acinar shadows), known to be highly correlated with cavitation and smear positivity, and reported to be present on chest radiograph in approximately 20% of adult PTB patients^16, 17^ was present in only 6.7% of subclinical PTB patients. Remarkably, normal chest radiographs were present in 17.5% of subclinical PTB patients. They have previously been reported in a proportion of adolescents and adults with primary PTB^16^, but we found that only 30% of our subclinical patients with a normal chest radiograph could be linked to recent TB contact and thus be considered to have primary PTB. Moreover, intrathoracic adenopathy, another, albeit less common, feature of primary PTB in adolescents and young adults, was rare, present in only 9 (3.1%) subclinical PTB patients, none of whom could be linked to recent TB contact.

That the CT scan was much more sensitive than the chest radiograph in the detection and characterization of both parenchymal disease and intrathoracic lymph node enlargement; cavitation (27.7% vs. 7.4%,), moderate to far advanced or miliary disease (42.6% vs. 19.2%), and lymph node enlargement (22.3% vs. 5.3%), was not surprising given the higher resolution of CT^19, 20^. However, the magnitude of the differences in the frequencies of features on CT scan versus chest radiograph were larger than has been previously reported for adult-type PTB. For example, with respect to cavitation and endobronchial spread, differences of 58% vs 22% (2.6 fold) and 98% vs. 24% (4.1 fold), respectively, have been reported in the past using high resolution CT [14], whereas we saw 3.7 fold and 9.1 fold differences, respectively, in these two features in subclinical patients. While this may in part reflect improvements in CT scan performance characteristics (e.g. collimation, temporal resolution), it may also reflect a proportionately greater limitation of the chest radiograph in subclinical disease. We are aware of only one other study that has reported on the CT scan findings in subclinical PTB^21^. This study was performed in South Korea and although their inclusion criteria for subclinical patients differed somewhat from our own (their patients were older aged [> 18 years vs > 14 years in our study] and may or may not have been culture-positive [all of our patients were culture-positive]), the frequencies of key findings were only slightly higher than those we report; cavitation 44.4%, “tree-in-bud” (endobronchial spread) 69.1%, and multiple lobe involvement (more extensive disease) (29.6%).

Early reports of CT scan findings in adult-type PTB suggested that endobronchial spread was present on CT scan in virtually all active PTB patients, with the exception of those with miliary disease and a small fraction (10%) of those with sputum smear-negative PTB, regardless of CT scan collimation^14, 22–26^. However, as noted above only 59.6% (28.6% of those with no lung parenchymal disease on chest radiograph) of our subclinical PTB patients had evidence of endobronchial spread on CT scan. While the presence of endobronchial lesions on high resolution CT may prove helpful in making a distinction between active and inactive PTB disease on chest radiograph^14, 23^, their absence clearly does not rule out active subclinical PTB disease. The pathologic correlates of lung parenchymal disease in that subset of HIV-seronegative, new-active subclinical PTB patients with no evidence of endobronchial spread on CT scan, would be of special interest^14, 27^.

The global pandemic of COVID-19, caused by severe acute respiratory virus 2 (SARS-CoV2), has rekindled interest in the question of asymptomatic PTB transmission. COVID-19 has shown that asymptomatic infection can be important in disease transmission^28^. In our subclinical patients smear-positivity and evidence of cavitation on chest radiograph, known predictors of the infectiousness of PTB, were uncommon^29–31^. However, cavitation and more extensive disease were much more common on CT scan. And others have found that these same features, along with a typical pattern of disease on CT scan, have been associated with smear-positivity^24, 26, 32^. Although most of our patients were smear-negative, we have previously shown that shorter times-to-culture positivity, an indicator of a higher bacillary burden or more metabolically active bacteria^33, 34^, and therefore, theoretically, a greater likelihood of transmission, were associated with more extensive disease on chest radiograph^6^. Almost certainly there is a radiologic/mycobacteriologic gradient of transmission risk within subclinical PTB source cases, as is known to be the case in PTB source cases in general. This same gradient may ultimately prove to be useful in determining the duration of treatment necessary to cure subclinical PTB^35, 36^.

Strengths of our study include the size of the cohort, the quality and completeness of the epidemiologic, radiologic and mycobacteriologic data, and the prospective interpretation of the radiographs and CT scans by multiple highly qualified readers. Limitations include the study’s retrospective design which meant that not all patients underwent a CT scan, thus raising a question about the representativeness of the sample. In support of its representativeness the chest radiographic features in those who had or had not been scanned were, for most part, comparable. The only exception was radiograph category, which likely reflected the greater probability that an atypical pattern would prompt the ordering of a CT scan. Some CT scans, specifically, those of the neck in patients whose chest radiograph had shown no abnormality below the main carina, only included the lung apices. In others,scanners and scan parameters such as collimation and enhancement, varied, though in this regard it is noteworthy that our inter-reader variability analysis suggested that there was little ambiguity about the findings. Finally, though most radiographs and CT scans were performed contemporaneously, this was not always so. Any changes that occurred in the interval separating the studies would compromise the comparison. These limitations could be overcome in a prospective study that included both CT scan and chest radiograph in all consenting subclinical patients, that used the same scanners and scan parameters in all patients, and that used a fixed, short, time interval between CT and radiographic imaging.

We conclude that when strictly defined, most subclinical PTB patients in Canada have minimal or no lung parenchymal disease apparent on chest radiograph. However, when CT scans are performed in the same patients, the chest radiograph may be shown to significantly under-detect the presence of key features of potential importance to transmission risk and treatment duration.

## Supplementary Information

Supplementary Information.

## Data Availability

The datasets used and/or analysed during the current study are available from the corresponding author on reasonable request.
