# Supplementary material for: A comparison of the chest radiographic and computed tomographic features of subclinical pulmonary tuberculosis
Source: Sci Rep. 2022 Oct 4;12:16567. doi: 10.1038/s41598-022-21016-7 (PMC9531232; doi:10.1038/s41598-022-21016-7)
Supplement: Supplementary file 1 — Supplementary Information. [file 41598_2022_21016_MOESM1_ESM.docx]

**Supplements:**

1. Data abstraction form, chest radiograph
2. Data abstraction form, CT scan
3. Extent of parenchymal disease criteria: US National Tuberculosis and Respiratory Disease Association

**Supplement 1.**

| **1** |  | **Category** | |  | |  |
| --- | --- | --- | --- | --- | --- | --- |
|  | |  | Typical □ | Atypical □ | | Normal □ |
|  |  |  | |  | |  |
| **2** |  | **Laterality** | |  | |  |
|  | |  | Unilateral □ | Bilateral □ | | Normal □ |
|  |  |  | |  | |  |
| **3** |  | **Cavitation** | | Yes □ | | No □ |
|  | |  | If Cavitary: | Single □ | | Multiple □ |
|  | |  |  | Diameter of Largest Cavity: ________ CM | | |
|  | |  |  | |  | |
| **4** |  | **Acinar Shadows:** | | Yes □ | | No □ |
|  |  |  | |  | |  |
| **5** |  | **Mediastinal or Hilar Lymphadenopathy** | | Yes □ | | No □ |
|  |  |  | |  | |  |
| **6** |  | **Extent of Disease:** | | Normal □ | | Minimal □ |
|  |  |  | | Moderately-advanced □ | | Far-advanced □ |
|  |  |  | |  | |  |
| **7** |  | **Volume Loss:** | | Yes □ | | No □ |
|  |  |  | |  | |  |
| **8** |  | **Pleural Thickening / Retraction** | | Yes □ | | No □ |
|  |  |  | |  | |  |
| **9** |  | **Pleural Effusion** | | Yes □ | | No □ |
|  |  |  | |  | |  |
| **10** |  | **Both PA and Lateral Views** | | Yes □ | | No □ |
|  |  |  | |  | |  |

Additional Comments

**Supplement 2.**

| 1. **Category:** Typical □ Atypical □ | | | 1. **Laterality:** Unilateral □ Bilateral □ | | |
| --- | --- | --- | --- | --- | --- |
| 1. **Centrilobular Nodule  or Opacity** | | Yes □  No □ | 1. **Tree-in-bud** | | Yes □  No □ |
| 1. **Poorly Defined Nodule** | | Yes □  No □ | 1. **Lobular (or more) Consolidation** | | Yes □  No □ |
| 1. **Bronchiectasis** | | Yes □  No □ | 1. **Volume Loss** | | Yes □  No □ |
| 1. **Bronchial Wall Thickening** | | Yes □  No □ | 1. **Cavitation** | | Yes □  No □ |
| **If Yes:** | Focal □  Generalized □ | | **If Yes:** | Single □ Diameter of  Multiple □ Cavity **_______** | |
| 1. **Mediastinal Lymph Node Enlargement** | | Yes □  No □ | 1. **Mosaic Pattern of Reduced Lung Attenuation** | | Yes □  No □ |
| 1. **Bronchovascular Distortion** | | Yes □  No □ | 1. **Mucus Impaction in Large Airway** | | Yes □  No □ |
| 1. **Emphysema** | | Yes □  No □ | 1. **Miliary Pattern** | | Yes □  No □ |
| 1. **Pleural Thickening / Retraction** | | Yes □  No □ | 1. **Pleural Effusion** | | Yes □  No □ |
| 1. **Extent of Disease** Normal □ Minimal □ Moderately-advanced □ Far-advanced □ | | | | | |

Additional Comments

**Supplement 3.**

1. **Normal** This classification applies to lesions that cannot be seen on the roentgenogram but are associated with positive cultures for *M. tuberculosis* complex.
2. **Minimal** lesions include those that are of slight to moderate density but do not contain demonstrable cavitation. They may involve a small part of one or both lungs, but the total extent, regardless of distribution, should not exceed the volume of lung on one side, which is present above the second chondrosternal junction and the spine of the fourth or the body of the fifth thoracic vertebra. The term minimal is not to be interpreted as minimizing the activity or hazards of the disease in this stage.
3. **Moderately advanced** lesions may be present in one or both lungs, but the total extent should not exceed the following limits: disseminated lesions of slight to moderate density that may extend throughout the total volume of one lung or the equivalent in both lungs; dense and confluent lesions that are limited in extent to one-third the volume of one lung; total diameter of cavitation, if present, must be less than 4 cm.
4. **Far advanced** is used to describe lesions that are more extensive than moderately advanced.
5. **Miliary** Diffuse micronodular pattern.

ss
